# Case of neonate with total intestinal hirschsprung disease managed with a proximal jejunostomy

**DOI:** 10.1016/j.intf.2025.100052

**Published:** 2025-04-03

**Authors:** Farhana Ali-Geiger, Michael Traynor, Lauren Baumann, Brian Bucher

**Affiliations:** a Department of Pediatrics, Division of Gastroenterology, Hepatology and Nutrition, University of Utah, USA; b Department of Surgery, Division of Pediatric Surgery, University of Utah, USA

**Keywords:** Hirschsprung, Total intestinal aganglionosis, Pediatric intestinal failure, Jejunostomy

## Abstract

**Background:**

Hirschsprung disease (HD) is a congenital disorder of the enteric nervous system, and its management will differ clinically depending on the degree of aganglionosis. Total intestinal HD (TIHD) is a rare variant, accounting for < 1 % of all cases, and is defined as aganglionosis of nearly the entire intestine with less than 20 cm of ganglionated small bowel past the ligament of Treitz (LOT). TIHD can be challenging to manage with high risk for chronic intestinal failure.

**Case report:**

Ours is a neonate confirmed pathologically to have total intestinal aganglionosis, surgically managed with a jejunostomy formed at 40 cm distal to LOT.

**Conclusion:**

Case of TIHD that has benefitted from a proximal diverting jejunostomy.

## Introduction

Hirschsprung disease (HD) is a congenital disorder characterized by the absence of ganglion cells in the myenteric and submucosal plexuses of the intestines [1]. Clinical management will differ depending on the degree of aganglionosis. The American Pediatric Surgical Association (APSA) Outcomes and Evidence Based Practice Committee (OEBPC) recommends standardizing nomenclature for the separate categories of HD. Near-total or total intestinal HD (TIHD) is a rare variant, accounting for < 1 % of all cases, defined as aganglionosis of nearly the entire intestine with < 20 cm of ganglionated small bowel past the ligament of Treitz (LOT) [2]. TIHD poses management challenges, with high risk for chronic intestinal failure and difficulty weaning off parenteral nutrition (PN). The European Reference Network for rare Inherited and Congenital Anomalies (ERNICA) is developing clinical consensus statements pertaining to total colonic aganglionosis and total intestinal aganglionosis in regard to surgical treatment, bowel function management, and long-term care [3]. Currently, there is no consensus statement on the most effective surgical reconstruction for total intestinal aganglionosis. However, near consensus suggests formation of jejunostomy at least 40 cm distal to LOT as the safest initial surgery, despite leaving a variable aganglionic segment of jejunum in continuity. We present a case of total intestinal aganglionosis not only for its rarity among forms of HD, but to highlight the treatment consideration for a proximal jejunostomy formed 40 cm distal to the LOT.

## Case report

The patient is a male infant born term via normal vaginal delivery. Following delivery, the infant began breastfeeding but shortly after developed bilious emesis. An abdominal x-ray was worrisome for dilated proximal small bowel, though a subsequent upper GI study was normal without evidence of malrotation or volvulus. The neonate had not yet passed meconium, and a follow up x-ray on day of life (DOL) 2 demonstrated slow progression of contrast with increasing gaseous distention, and a barium enema revealed a microcolon. An exploratory laparotomy on DOL 3 found a transition zone in the mid ileum that did not appear to have an extrinsic cause, raising concerns for long segment HD. The transition zone was resected with creation of an ileostomy and mucous fistula. Histopathology from the resected ileal specimen and rectal suction biopsy revealed absence of ganglion cells and myenteric plexus; PHOX2B immunostaining was negative, and there were absent positively stained calretinin nerve fibers. On DOL 8 he was taken back to the OR for intraoperative leveling biopsies to determine the transition zone. There was 95 cm of small bowel from LOT to the ileostomy. Multiple full-thickness small intestinal biopsies were obtained, with frozen section revealing rare ganglion cells at 15 cm distal to the LOT. Given the extent of disease, we elected to wait for final pathology to discuss definitive management, while a gastrostomy tube (G-tube) was placed to supplement future enteral feeds. Final pathology was negative for submucosal ganglion cells with absent myenteric plexus in all but one of the specimens - the specimen at 15 cm – confirming the diagnosis of TIHD. He was medically managed for the time being with total parenteral nutrition (TPN) and kept nil per os. A Non-Syndromic Hirschsprung Disease (HD) genetic panel was sent revealing a heterozygous variant of unknown significance in the *RET* gene at C.2870 C>G.

After a multidisciplinary care conference with the family, it was proposed to the surgical team to divert the existing stoma more proximally at 40–50 cm from LOT. The neonate was ultimately taken back to the OR at DOL 58 for re-exploration. He was found to have a natural transition point at 40 cm from LOT. Additional leveling biopsies of colon confirmed no ganglion cells present. The small intestine was resected up to the transition point with creation of an end jejunostomy at 40 cm from LOT, a total abdominal colectomy was performed. A tunneled central venous catheter (CVC) was also placed for long term PN. He recovered from the surgical procedure, with initiation of trophic tube feeds on post operative day (POD) 5 using breastmilk while TPN provided full fluid and energy needs. He was switched to continuous tube feeds for better tolerance, initiated at 5 mL/kg/day (1 mL/hr) with stable ostomy output < 20 mL/kg; due to significant oral aversion, oral feeds were non-contributory to his enteral goals. While he did have mild cholestasis (highest direct bilirubin 1.7 mg/dL) prior to last operation, bilirubin improved and normalized with enteral feedings within couple weeks. He was discharged home on POD 20 with outpatient follow up with intestinal rehabilitation clinic and surgery.

Outpatient feeding advancements were done slowly and monitored closely, advancing rate by 1–2 mL weekly based on ostomy output, feeding tolerance, weight gain, and labs (monitoring serum electrolytes and urine sodium). His total energy needs from enteral and parenteral nutrition were kept 1.2x the Recommended Dietary Allowance (RDA) for age to maintain appropriate weight gain velocities. By 6 months, he transitioned to elemental formula once breastmilk was no longer available, and jejunostomy outputs remained between 30 and 40 mL/kg as enteral feeds were increased. Now at one year of age, the child has progressed on enteral feeds with G-tube feeds of 28 mL/hr on Neocate Infant 20 kcal/oz, with stable jejunostomy output < 40 mL/kg without the need for anti-diarrheal agents, and with adequate growth (weight-for-age z-score −0.9, weight-for-length z-score −1.0, mid-upper-arm circumference z-score −1.2). He does not have issues with cholestasis or PN-associated liver disease. He has been able to wean down on parenteral support with TPN consolidated to 16 hours a day, providing ∼75 % of his energy needs. He did incur complications with his central line at 8-months of age with his first, and thus far only, line-associated blood stream infection that necessitated line removal due to inability to clear the infection; a new CVC was placed with prophylactic lock therapy implemented using sodium bicarbonate.

## Discussion

Total intestinal aganglionosis is the rarest and most severe form of HD. Given the heterogenicity in the literature and variability in definitions, the APSA OEBPC has recommended standardizing HD nomenclature with TIHD defined as ganglionated bowel < 20 cm distal to the LOT. Up until a few decades ago, it was considered to be a uniformly fatal condition [4], [5], [6]. However, survival has improved in recent years with the advent of PN, advances in surgical techniques, and small bowel transplantation [7], [8], [9]. In 2024, ERNICA released consensus statements on the management of total colonic and intestinal aganglionosis. For the management of total intestinal aganglionosis, the panel reached consensus on the recommendation for early referral to an expert intestinal rehabilitation group to prevent complications related to long-term PN. For surgical management, the panel reached near-consensus for a jejunostomy be formed at least 40 cm distal to the LOT; consensus was not reached as some members favored a more distal stoma. It was the panel’s opinion that the distal diverted bowel be resected in a timely manner given the risks of enterocolitis, though some may choose to leave the aganglionic intestine in-situ until symptoms of enterocolitis develop, or to preserve the intra-abdominal domain for future intestinal transplantation. There was no consensus on the preference for a G-tube at time of jejunostomy formation; some felt that enteral feedings would not be a problem and that it should be encouraged to decrease risks of intestinal failure-associated liver disease [3].

In our TIHD case, the creation of a jejunostomy at 40 cm from LOT aligns with ERNICA’s surgical recommendations. Our case provides promise into an optimal surgical strategy in the challenging management of TIHD. The surgical approach of forming a stoma at a site distal to the aganglionic segment has been previously published, where in Kimura et al., the jejunostomy is created at 30 cm distal to LOT to prevent recurrent enteritis [10]. In their cohort, survival amongst the infants with TIHD were those with a jejunostomy 30–35 cm from LOT who also had an extended myectomy-myotomy performed. It is postulated that preserving aganglionic bowel has possible risks for HD-associated enterocolitis (HAEC). Despite HAEC being considered one of the most fatal complications in HD with variable mortality ranges, there have been reports of lower rates of HAEC occurring in TIHD than other forms of HD [11]. Patients with aganglionic proximal jejunal segment left in situ have demonstrated preserved intestinal passage without increased obstructive episodes, and some enteral tolerance permitting reductions in parenteral nutrition [12].

Despite the dismal outcomes reported in literature, our patient has had a meaningful quality of life with few complications. With intestinal aganglionosis, lack of normal bowel to support full enteral feedings renders chronic PN necessary with preserved central venous access. Severe PN-associated complications such as cholestasis and liver disease, along with compromised vascular access, will accentuate the problems these patients face including stoma complications, sepsis and enterocolitis, and electrolyte/fluid imbalances. At this time, our patient has been tolerating enteral feeding advancements with concurrent decreases in parenteral support, and with diligent care of the central line. While a small bowel transplantation may represent the only realistic option for definitive treatment, we have hope that such a need would be in the far distant future, once the patient has run out of alternatives.

## Patient consent

Parental consent was obtained for this case report

## Ethical clearance

Not required

## Funding

This research did not receive any specific grant from funding agencies in the public, commercial, or not-for-profit sectors

## CRediT authorship contribution statement

**Ali-Geiger Farhana:** Writing – original draft, Conceptualization. **Traynor Michael:** Writing – review & editing. **Baumann Lauren:** Writing – review & editing. **Bucher Brian:** Writing – review & editing.

## Declaration of Competing Interest

The authors declare that they have no known competing financial interests or personal relationships that could have appeared to influence the work reported in this paper.
